# How Knowledge Mapping Can Bridge the Communication Gap Between Caregivers and Health Professionals Supporting Individuals With Complex Medical Needs: A Study in Fragile X Syndrome

**DOI:** 10.3389/fpsyt.2021.731011

**Published:** 2021-11-24

**Authors:** Karen Kelm, Francois V. Bolduc

**Affiliations:** ^1^Department of Pediatrics, University of Alberta, Edmonton, AB, Canada; ^2^Department of Medical Genetics, University of Alberta, Edmonton, AB, Canada; ^3^Neuroscience and Mental Health Institute, University of Alberta, Edmonton, AB, Canada; ^4^Women and Children Health Research Institute, University of Alberta, Edmonton, AB, Canada

**Keywords:** Fragile X syndrome, care mapping, communication, mental model, medical complexity, knowledge mapping, concept mapping

## Abstract

The challenges of caring for children with complex health needs, such as intellectual disability (ID) and autism spectrum disorder (ASD), are multiple and experienced by both caregivers and health professionals. Fragile X syndrome (FXS) is the most common single gene cause of ID and ASD, and provides a pertinent model to understand these complexities of care, as well as the communication challenges experienced between caregivers and healthcare professionals. In recent years both caregivers and healthcare professionals have recognized the need for enhancing communication both in clinical and research settings. Knowledge mapping has emerged as a tool to support quality communication between team participants. Here we review how differences in mental models, as well as challenges related to health literacy and knowledge transfer can have an impact on communication. Next, we present different knowledge mapping approaches used in complex situations, with a focus on concept maps and care maps. Finally, we highlight the potential benefits and limitations of mapping to improve communication issues related to caring for individuals with FXS and potentially other neurodevelopmental disorders (NDDs).

## Introduction

### Neurodevelopmental Disorders Represent a Prevalent Case of Medical Complexity

Among the many conditions associated with pediatric medical complexity, neurodevelopmental disability (NDD) is one of the most notable as they affect 3–18% of the world's population (1–7). NDDs include conditions such as Attention Deficit/Hyperactivity disorder (ADHD), learning disability, intellectual disability (ID), and autism spectrum disorder (ASD). Most individuals with an NDD not only have multiple core symptoms linked to their condition but also experience related (comorbid) conditions, and as such are usually followed by a large team of health and education specialists (8). Having multiple partners involved in care coordination can create challenges in communication. Quality communication in healthcare is essential to ensure a high standard of care; poor communication often results in unmet family needs, dissatisfaction in care, and potentially even medical errors or needless interventions (9–11).

The complexity in needs and services for individuals with NDDs tasks caregivers and healthcare professionals with enormous responsibilities (12). For caregivers, the impact of managing their child's multiple health complexities, the lack of training to provide expert care, and grief can contribute to an overall feeling of helplessness and exhaustion (13, 14). Additionally, caregivers encounter financial challenges and can struggle with depression (15–17). Caregivers often become a “manager” for their child: advocating for and coordinating their child's care, managing communication between healthcare and social providers, implementing therapeutic recommendations, and maintaining their child's medical health information and records (16–18). For healthcare professionals, children living with multiple health complexities belong to a unique population that demands ongoing continuous care and utilizes a disproportionate amount of healthcare services (12, 13, 16, 17, 19–22). In addition, healthcare professionals can be required to provide care that often extends beyond their capacity due to limitations in skills, psychosocial support, opportunities for continuing education, or lack of resources (13, 23, 24).

### Fragile X Syndrome as a Model for Complex Care in NDD

In this review we focus on Fragile X syndrome (FXS) as it is the most common single gene cause of ASD and ID. More importantly, FXS illustrates the complexity of symptoms and the challenges in communication between caregivers and healthcare professionals seen in most NDDs. Indeed, individuals with FXS present clinically with a wide spectrum of symptoms and comorbid conditions, including core cognitive and adaptive function challenges (25), speech delay (26, 27), autism spectrum traits (28–31), sleep issues (32–34), challenging behavior (35), anxiety (36), and mental health issues (37). FXS is also an excellent model for understanding changing needs over a lifespan as, like most NDDs, it is a lifelong condition where the health, educational, and social needs of individuals evolve over time, making care even more complex and challenging for parents and health professionals (38). As a child diagnosed with FXS enters each life stage, new symptoms and behaviors can appear while existing ones can potentially intensify (39). As the child transitions to adult services, parents take on the responsibility of initiating and transferring services and funding from youth to adult programs (38, 40–44). There is no gold-standard model for transition from pediatric care to adult care, making the transition to adult services a struggle for families to know what to do. Limited access to services can create anxiety and decreased quality of life for both parents and individuals living with FXS (38, 45, 46).

Finally, FXS highlights the importance of considering the family unit when assessing complexity in care and communication. In FXS, mothers can have a range of genetic variants from carriers of a pre-mutation which does not manifest as FXS but can lead to biological (e.g., early menopause) and psychological (e.g., anxiety and depression) challenges to full mutation with FXS symptoms, which need to be considered during the communication process.

Through a review of the literature, this mini-review summarizes the key components impacting communication between caregivers and healthcare professionals, and explores the use of knowledge mapping as a tool to strengthen quality communication. Our goal is to evaluate these challenges in communication through the lens of care needed to support individuals with FXS. We will discuss: (1) the key components impacting communication between caregivers and healthcare providers in FXS and other medically complex situations, (2) ways to visually represent and share complex information, known as knowledge mapping, as a method to enhance communication, and (3) how mapping has been shown to improve communication in complex situations.

Our central hypothesis is that communication challenges between caregivers and health professionals may be improved with the implementation of knowledge mapping.

## Methods

In order to better understand how to improve the quality of communication between caregivers and healthcare professionals, we performed a 2-step literature review using a pragmatic approach. The most relevant papers are cited. First, we conducted a general literature review in order to identify the components impacting communication between caregivers and healthcare professionals in the context of children with complex needs. We used the following keywords to search English language text from PubMed and Google Scholar with no limitation on time period: “*healthcare professionals, physicians, communication, caregivers, parents, patients, pediatric healthcare, complex medical needs, neurodevelopmental disabilities, NDD, intellectual disability, ID, Fragile X syndrome, FXS, attention deficit/hyperactivity disorder, ADHD, autism spectrum disorder, and ASD.”* We included both original articles and reviews, and identified 68 papers. We reviewed those papers and identified converging themes centered around “*mental model, health literacy, knowledge transfer, beliefs, perspectives, mapping, concept mapping, and care mapping*.”

Next, we focused on mapping methods for concepts relevant to complex situations and healthcare, searching for articles discussing “*communication, mapping, knowledge mapping, mental models, concept mapping, and care mapping.”* We identified 46 papers and 3 books which were used to prepare the themes of review.

## Results

### Components Impacting Communication Between Caregivers and Healthcare Professionals in FXS and Other Medically Complex Situations

While there are many factors leading to communication challenges in complex medical situations, we found that a key aspect resided in the concepts and categories individuals have developed over time. Challenges in communication present themselves when individuals have conflicting concepts and categories. This is known as lack of coherence in mental models. Two factors contributing to the difference in mental models were the base knowledge of individuals, also known as literacy, and their ability to exchange information, referred to as knowledge transfer. We discuss below those 3 interlinked concepts.

#### Coherence in Mental Models of Caregivers and Healthcare Professionals Drive Quality Communication

**Figure 1:**
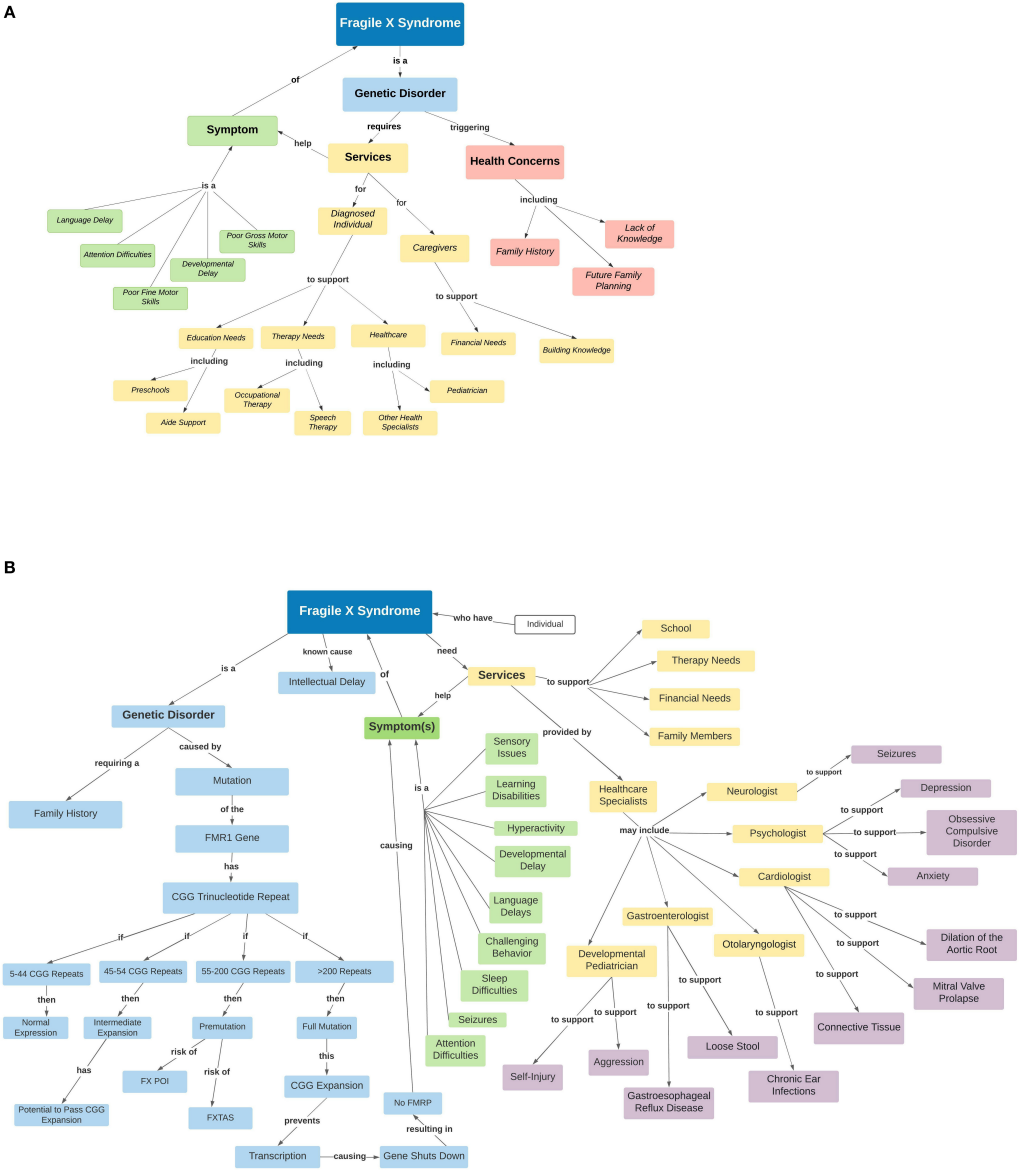
Mental models vary between caregivers and healthcare professionals supporting individuals living with FXS. Concept maps representing the mental models of Fragile X syndrome symptoms and associated conditions (comorbidities) from a caregiver's perspective **(A)** and a healthcare professional's perspective **(B)**. This visual representation shows how mental models of FXS can vary between caregivers and health professionals, and may overlap in others (healthcare for instance). **(A)** The mental model of a caregiver may include concepts related to personal concerns outside of healthcare and a general understanding of the health system. **(B)** On the other hand, the mental model of a healthcare professional may only focus on the concepts related to healthcare more specifically, with a bias toward the consideration of their specialty. Legend of abbreviations used in the figure: FMR1, Fragile X mental retardation 1 gene; FXPOI, Fragile x Premature ovarian insufficiency; FXTAS, Fragile X tremor and ataxia syndrome.

A person's understanding of concepts and their relation to other concepts is formed by past experiences, education, and perceived knowledge, and is referred to as a mental model (47). Mental models are also influenced by multiple factors including family status, cultural beliefs, education, literacy, and goals (48, 49). Mental models play a significant role in an individual's decision making and behavior, as well as communication. In Figure 1 we use a concept map as a tool to visually represent a caregiver's mental model (**1A**) and a healthcare professional's mental model (**1B**) to show how mental models can vary between caregivers and healthcare professionals supporting individuals living with FXS.

In healthcare, research in obesity, nursing, clinical teamwork, and oncology has identified how understanding individual mental models has the ability to increase efficiency in team performance when mental models are shared (48, 50–53). It also showed that shared understanding of an individual's mental model prevents communication errors and opens the door for effective communication, collaboration, and navigation within the health system (50, 52). Researchers have also studied the mental models of patients with varying mental health conditions such as depression, obsessive compulsive disorder, bipolar disorder, and schizophrenia to better understand the condition but also clinical care (54–59).

Mental models are variable between individuals, an important issue to acknowledge for optimal communication. Surveys of caregivers supporting individuals with FXS showed an important diversity in perceptions of the impact of a FXS diagnosis, burden of caring for a child with FXS, and decision making (60–63). Professionals working in healthcare present with variability in mental models too. When comparing the mental models of obesity between healthcare professionals and policymakers, Sturgiss et al. identified a clear distinction between each group (48).

#### Health Literacy Contributes Significantly to Mental Model

A key building block of a mental model is health literacy, which is defined as “the ability to access, understand, evaluate, and communicate information in a way to promote, maintain and improve health in a variety of settings across the life-course” (44). While limitations in space do not allow for a full review of the large number of studies in health literacy (64, 65), we highlight below how literacy impacts communication. Indeed, a caregiver's health literacy has a direct impact on the health of their children (66, 67). Caregivers with low health literacy lack the knowledge to associate appropriate health services with specific care needs, and experience a disconnect in communication and flow of information with healthcare professionals (13). Interestingly, a caregiver's health literacy has the potential to evolve with time through trial and error, extensive research, online resources, peer recommendations, and analysis of journal articles (68, 69), which can in turn lead to evolution of the mental model of that individual. In FXS, as for many other medical conditions, another aspect to consider is that a large amount of information must be gained rapidly. For instance, a caregiver whose child is diagnosed with FXS will need to learn about FXS but also linked diagnosis related to carrier status (70).

Additionally, when a caregiver assumes their healthcare provider has a lack of health literacy around their child's diagnosis, most caregivers will hold a negative attitude toward that healthcare provider (71). This issue is multiplied when considering rarer conditions. Often with limited exposure to FXS or genetic disorders, non-specialist healthcare providers have been shown to have limited knowledge about genetic testing in general as well as FXS (72, 73).

#### Knowledge Transfer to Improve Coherence of Mental Model

As mentioned above, mental models can evolve over time due to a growth in health literacy. This happens in large part due to knowledge transfer between caregivers and health professionals (48). While mental models are updated by knowledge transfer, knowledge transfer is also influenced by mental models. Indeed, researchers explained that when mental models of a specific topic do not align, knowledge transfer becomes challenging as “neither group can conceptualize the others' viewpoint” (48). During the knowledge transfer process, it is important to use terminology known and understood by families (74, 75). A successful knowledge transfer process involves information to be collected, evaluated, and organized, then shared using relevant language.

When caregivers cannot accurately communicate in a language understood by healthcare professionals, healthcare professionals struggle to fully understand how they can make an impact on the care of their patients and the lives of their caregivers (13). For healthcare professionals, it is important to assess the emotional state of the caregivers. For example, families having just received a diagnosis of FXS for their child identify this as a very distressing time (76). Inefficient knowledge transfer will often lead caregivers to be unsure when to seek help for their child, to have feelings of inadequacy, to lack awareness of their child's condition, to have repeated interactions with healthcare professionals, to be unable to make decisions, and to be unaware of who to reach out to for care (13).

### Knowledge Mapping: A Way to Represent and Share Complex Information Visually

Due to the complex, dynamic, and often incomplete information being shared between caregivers and healthcare professionals, finding an efficient way to present and organize communication is essential to quality care. Fortunately, cases in other fields have shown that knowledge mapping is a valuable tool to efficiently exchange concepts, as well as simplify verbal and written communication (77). In general, knowledge mapping is a tool used to support quality communication by visually representing information (78, 79). In our research we focused on using knowledge mapping to represent individuals' knowledge, beliefs, and perspectives. Several mapping methods are available and can be used in multiple applications depending on the need. These include: service blueprint, customer journey map, experience/care map, concept map or mental model diagram, and spatial map (80). We focused on two types of mapping tools: **(A)** concept map and **(B)** care map as they have been used most frequently to represent mental model and clinical care, respectively.

#### Concept Map

A concept map can visually represent the mental model of an individual by showing the relationships between concepts and ideas in a hierarchical manner (78). Concept maps link core concepts to related concepts (48). In Figure 1 we illustrate an example of using concept maps to represent a sample mental model of FXS for a caregiver and healthcare professional. When comparing the concept map of the caregiver and the healthcare professional it is clear where the mental models of care needed to support an individual with FXS differ. Concept maps may constitute an initial step in communication between caregivers and health professionals, allowing each “team player” to express their perceived understanding of a condition. When communicating about the needs of an individual diagnosed with NDD, including FXS, acknowledging the diverse mental models of each care provider is essential as there is an important variation between individuals in the severity of not only core symptoms, but also associated conditions (comorbidities) as mentioned above.

#### Care Map

**Figure 2:**
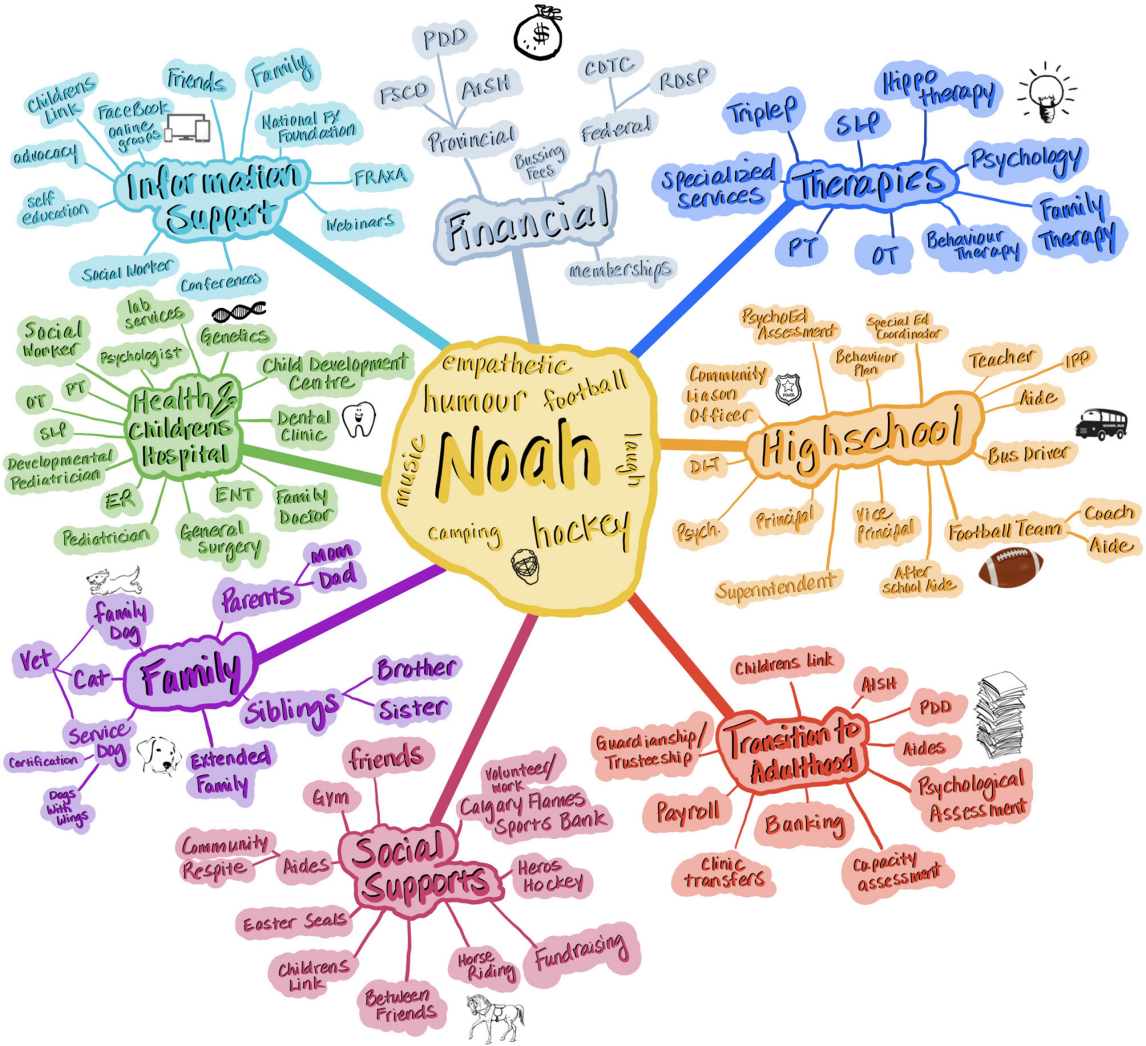
Care maps help to display the multiple entities related to individuals with FXS. A care map captures the multidimensional aspect of care needs and is organized around a given individual. Use of pictures, colors, and shapes can help to involve youth in the development of the care map while identifying priorities over time. Care maps are often color coded to provide visual distinction between fields. Note that overlap between providers becomes more apparent and allows for collaboration. For instance, occupational therapy could be provided in school and community. Legend of terms used in the figure: PDD, Persons With Developmental Disabilities (Provincial funding program in Canada); FSCD, Family Support for Children with Disabilities (Provincial funding program in Canada); AISH, Assured Income for the Severely Handicapped (Provincial funding program in Canada); CDTC, Child Disability Tax Credit (Federal funding program in Canada); RDSP, Registered Disability Savings Plan; SLP, Speech Language Pathologist; PT, Physical Therapy; OT, Occupational Therapy; ER, Emergency Room; ENT, Ear, Nose, and Throat Specialist; IPP, Individual Performance Plan; DLT, Diverse Learning Teacher.

A care map is adapted from the principles of a mind map, which is a more general map of concepts related to a topic (78, 81). These maps are designed in a radial structure with the patient at the center and peripheral topics related to the individual's care needs for whom the map is made. Pictures and/or words are used to organically represent a topic. A care map for an individual with FXS shows the multiple and diverse needs (related to all comorbidities) in their life (example in Figure 2). As can be seen by comparing Figure 2 to Figure 1, the care map is more applied and actionable than the concept map and shows how different service providers may overlap from different sectors (school, hospital, outpatient clinic, etc.). As such, we propose that the care map may be used downstream of the concept map in the optimal management of a patient. As Gavin points out, a picture is worth more than a thousand words, indicating that the visual of a care map delivers a powerful message of not only the complexities in care, but draws out the privilege, or lack thereof, some families may experience within the healthcare system (82). One family's map may identify multiple systems of care and services that may not be accessible to others due to location, knowledge of the services, diagnosis of the child, finances, language, or citizenship.

### Benefit of Knowledge Mapping in Complex Medical Situations

Research on care coordination correlates the use of: (**A)** concept mapping and (**B)** care mapping to an increased level of communication between healthcare providers and caregivers, as well as between healthcare providers themselves (19, 74, 75, 83). Furthermore, in healthcare mapping can promote health literacy, build and organize an individual's mental model, and enhance knowledge transfer (78, 84).

#### Impact of Concept Mapping

Concept maps are crucial as they allow identification of blind spots between caregivers and health professionals involved. This is important as increasing healthcare specialization, but also diversity in caregiver perspectives leads to further differences in mental models (85). Concept maps, by putting individual mental models in the open, allow people to acknowledge differences in perspective, personal bias, and lack of awareness in some aspects of a concept which are more remote to one's experience. Concept maps have been shown to be core to efficient knowledge sharing (86), education, and enhanced problem solving (87).

Mental model representation with concept maps allows researchers to group results and provide higher order analysis (48). We also propose that teaching programs for both caregivers and healthcare professionals, as well as health policies and community resources, would be made more impactful by considering mental models.

#### Impact of Care Mapping

There are multiple advantages to using a care map as well. Care maps increase the level of communication between caregivers and healthcare providers, as well as between healthcare providers themselves, by increasing the level of engagement and reciprocal communication while also reducing the barriers in status (19, 74, 75). Care maps have the potential to give families a voice to express not only their child's needs, but their needs as a caregiver (19, 74, 75). This enhanced engagement also comes from developing a care map (17) and the empowerment of families by giving them an opportunity to share their voice and express preference and priorities around their child's needs and care concerns, hopes, and caregiver goals (19).

A care map can incorporate pictograms (Figure 2) having the potential to involve children in their care and provides the opportunity to prioritize the interventions that are most important to them. According to Adams et al., care mapping can be seen as a universal language between healthcare providers (19). A care provider does not need to know a patient personally to understand the meaning and information reflected in a care map. In addition, there are educational opportunities offered by evaluating the information presented in a care map (16).

Care mapping identifies the importance of forming relationships between all participants in the decision-making process (48) and is an effective tool to develop empathetic relationships within a limited time frame, build partnerships, and support collaboration (19).

Care mapping provides the opportunity for individuals to expand their existing knowledge by providing new ideas and viewpoints, in turn enhancing their mental models (78).

## Discussion

Our literature review investigated the challenges of communication between caregivers and healthcare providers caring for individuals living with complex health needs, including NDD and more specifically FXS. We found that one's understanding of the condition of their child/patient, referred to as the mental model, was key in driving decisions, actions, and communication. We showed how mental models vary over time between individuals, and how health literacy and knowledge transfer contribute to leveling those differences in mental models.

We then discussed knowledge mapping methods which could be used to better communicate information between healthcare providers and caregivers. We propose that different knowledge mapping methods be combined considering their different outputs. We suggest that concept maps be used as a tool to represent one's mental model of a given topic. We showed that concept maps can identify a shared understanding and limit misunderstanding. We discussed multiple case studies that identified how crucial it is to recognize individual mental models, not only to facilitate quality communication but also for compatible team functioning. Next, we discussed how the complex care team caring for individuals with NDD and FXS could be better coordinated when presented with a visual representation of information like a care map. We also highlighted how developing the care map together with healthcare professionals generated engagement and empowerment for caregivers.

Further studies will be required to assess the cost effectiveness of these different mapping methods in the context of NDD. Moreover, while in theory very useful, we noted that most research on mapping was performed in specialized clinical or even research environments, which are not standard for most individuals with NDD. We therefore wonder about the uptake of care mapping and concept maps in clinical setups beyond specialized programs. Furthermore, studies investigating the internal and external validity of care and concept maps will be important to determine their effectiveness across patients, or conditions, or stages of life for individuals with NDD. Finally, considering the important changes over the lifespan of individuals with NDD, the changes in literacy for caregivers and health professionals over time, it remains unclear how long a map will be useful before becoming in itself a barrier to communication. More research into when and how maps will need to be updated will have to be done in the future.

Altogether, we hope our review will generate interest into developing mapping approaches to improve communication between healthcare providers and caregivers but also for any individual involved in each individual's team.

## Author Contributions

KK and FB performed the literature review, co-designed the project, and co-wrote the manuscript. All authors contributed to the article and approved the submitted version.

## Funding

The funding for this project comes from CIHR and NSERC.

## Conflict of Interest

The authors declare that the research was conducted in the absence of any commercial or financial relationships that could be construed as a potential conflict of interest.

## Publisher's Note

All claims expressed in this article are solely those of the authors and do not necessarily represent those of their affiliated organizations, or those of the publisher, the editors and the reviewers. Any product that may be evaluated in this article, or claim that may be made by its manufacturer, is not guaranteed or endorsed by the publisher.
